# Does the Post‐Natal Social Environment Influence Cognitive Development in a Social Gecko?

**DOI:** 10.1002/ece3.71560

**Published:** 2025-06-17

**Authors:** Birgit Szabo, Eva Ringler

**Affiliations:** ^1^ Division of Behavioural Ecology, Institute of Ecology and Evolution University of Bern Bern Switzerland; ^2^ Centre for Research on Ecology, Cognition and Behaviour of Birds University of Ghent Ghent Belgium

**Keywords:** behavior, cognition, developmental plasticity, fearfulness, reptile, squamate

## Abstract

The drivers of differences in cognitive ability, within as well as across species, remain a debated “hot‐topic” in animal cognition. Current hypotheses link variation in sociality, ecology, and more generally, environmental challenges to differences in cognition. Research supporting the social intelligence hypothesis, which states that cognition evolved to deal with the challenges of interacting with conspecifics, is largely focused on highly social mammal and bird species, limiting our ability to evaluate the general applicability of the hypothesis. Furthermore, it is unclear whether the social environment facilitates cognitive development or if selection acts against individuals with low cognitive abilities in social groups. Unfortunately, developmental studies which can reveal the causal link between early life experiences and cognitive development are scarce. Therefore, we aimed to test the effect of the early post‐natal social environment on the development of behavior and cognition in a social lizard, the tokay gecko ( *Gekko gecko* ). Our results show that the early social environment influenced the development of boldness and the variation in space neophobia and associative learning. We discuss our findings in the light of the social intelligence hypothesis, taking into account the facultative social nature of our study system.

## Graphical abstract

The generality of the link between the social environment and the development of cognition is not well understood on a broad taxonomic scale. We utilize developmental behavioral plasticity to test the causal link between the early social environment and the development of non‐social cognitive skills in the facultative social tokay gecko ( *Gekko gecko* ). We find that social deprivation affects the development of boldness, space neophobia, and associative learning. Our results demonstrate long‐term effects of early social experiences and confirm the suitability of lizards as models to test this link.

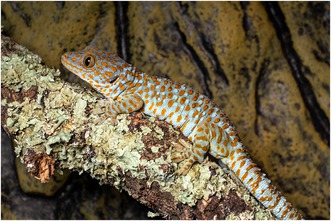

## 1 Background

Cognition is a general term for all neural processes by which individuals collect, retain, process, and use information gathered from their environment through the use of exploration, exploitation, or evasion that can lead to changes in behavior with the potential to increase survival and fitness (Shettleworth 2009; Lyon 2020). How cognition evolves, what causes differences in cognition, and what the consequences of these differences, are still some of the most intensely debated topics in the field. A number of hypotheses have been proposed connecting sociality (social intelligence hypothesis, Humphrey 1976; Jolly 1966; Chance and Mead 1953; Machiavellian intelligence hypothesis, Byrne and Whiten 1988; social brain hypothesis, Dunbar 1998), ecology (ecological intelligence hypothesis, Rosati 2017) or more generally challenges in the environment (cognitive buffer hypothesis, Sol et al. 2021) with enhanced cognition. A recent meta‐analysis has found general support for the social intelligence hypothesis across inter‐ and intra‐specific studies, as well as developmental studies (Speechley et al. 2024). This hypothesis links the demands of social interactions to the development of enhanced cognitive abilities to deal with the associated challenges of tracking individuals and maintaining social relationships across contexts and time (Humphrey 1976; Jolly 1966; Chance and Mead 1953). However, research is still biased towards mammals and birds, substantially limiting our ability to generalize the existence of this link across taxonomic groups (Speechley et al. 2024). Therefore, an increase in taxonomic breadth is urgently needed to develop a broad concept of the factors leading to differences in cognition both within and across species.

Research aiming to understand how group living is linked to cognitive ability, either through developmental effects or natural selection, has taken three broad directions: (1) large projects comparing cognitive abilities across species with varying degrees of sociality (e.g., Borrego and Gaines 2016; Devaine et al. 2017; MacLean et al. 2013, 2014), (2) intraspecific studies that compare cognition across individuals living in differently sized groups or groups with varying social complexity in the wild (e.g., Berhane and Gazes 2020; Ashton et al. 2018; Wascher 2015) and (3) controlled developmental studies (e.g., Bannier et al. 2017; Munch et al. 2018; Riley et al. 2017, 2018; Schäble et al. 2007; Zhang 2017). Developmental studies measure developmental plasticity, the development of variation in phenotypic traits resulting from different environmental conditions that are linked to differences in survival and reproductive success (Eastwood et al. 2023; Holloway 2002; Lee et al. 2013; Lindström 1999; Uller 2008). Developmental studies are a powerful tool to understand how group living might have shaped the evolution of cognition, as they can uncover the causal link between the social environment and cognitive abilities through developmental effects (e.g., Chapman et al. 2008; Meagher et al. 2015; Schrijver et al. 2002; Taborsky 2017; Toyoshima et al. 2018). The effects of the early social environment, especially social deprivation on the development of cognition, have been studied in a broad range of taxa from mammals to fish (e.g., Bannier et al. 2017; Munch et al. 2018; Riley et al. 2017, 2018; Schäble et al. 2007; Zhang 2017). However, only some of these studies found an early life effect on cognition, mostly showing a negative effect of social deprivation (i.e., social isolation or parental deprivation; e.g., Meagher et al. 2015; Toyoshima et al. 2018; but see e.g., Lévy et al. 2003; Riley et al. 2017, 2018). A potential reason for the mixed results might be a weak effect of social interventions, as identified in a meta‐analysis on the impact of environmental factors on learning abilities (Lambert and Guillette 2021). The existence and direction of the effects of sociality might depend on the social system of the tested species, as well as the cognitive trait tested (Lambert and Guillette 2021; Lévy et al. 2003; Speechley et al. 2024). Consequently, it is necessary to investigate the effects of the early social environment on the development of a large range of cognitive abilities, as well as in a large range of species expressing diverse sociality to gain a comparative evolutionary perspective (Ward and Webster 2016).

Various forms of group living (e.g., eusociality, cooperative breeding, fission fusion societies, long‐ and short‐term family groups) have evolved across all major vertebrate groups as well as in invertebrates (Rubenstein and Abbot 2017). However, research investigating the social intelligence hypothesis has traditionally focused on primates, rodents, and passerine birds (Speechley et al. 2024). Especially, studies in reptiles are lacking, hampering not just our understanding of the general applicability of the social intelligence hypothesis but also our understanding of what environmental factors influence reptile cognition. For instance, De Meester et al. (2019) found evidence that solitary squamates (lizards, snakes and worm lizards) have larger brains, a frequently used proxy for cognitive ability (e.g., Benson‐Amram et al. 2016; Lefebvre et al. 2004), compared to social species, contradicting the social intelligence hypothesis. To the best of our knowledge, no intraspecific studies comparing adult individuals across group sizes (like Ashton et al. 2018) have been done in reptiles so far, but three developmental studies have looked at how early social life influences cognition in two species of lizards. Tree skinks (*Egernia striolata*), a group‐living species, showed similar social and spatial learning ability regardless of whether they were reared alone or with a conspecific (age matched social partner; Riley et al. 2017, 2018). While in the White's skink (*Liopholis whitti*), a closely related group‐living species with a similar social structure, offspring raised with their mother performed better in a learning task (Munch et al. 2018). Consequently, due to the limited number of studies, a large gap is still present as to how different expressions of sociality might be linked to differences in cognition and what types of cognitive processes are affected. Facultative social species, such as reptiles, provide a powerful comparative model and a chance to look into the benefits of cognition as they might have occurred in early forms of group living.

**FIGURE 1:**
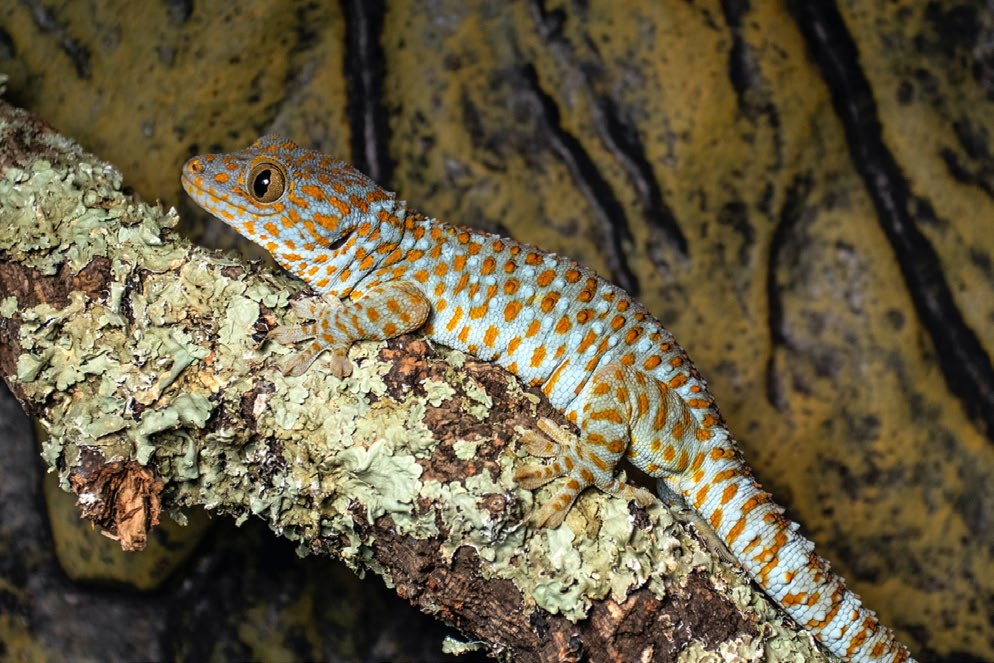
Adult, female captive bred tokay gecko ( *Gekko gecko* ) on a branch. Picture by Francesca Angiolani.

Therefore, the aim of this study was to test the effect of early post‐natal social deprivation on the expression of behavior and cognition in the tokay gecko ( *Gekko gecko* ; Figure 1) to understand if the simple social interactions that lizards show have an effect on the development of cognition similar to what has been found in other taxa (e.g., primates: Zhang 2017). Tokay geckos are a social lizard species that form pairs and family groups with biparental care (Grossmann 2007). Adults provide care to their eggs as well as offspring after hatching. Offspring stay with their parents until sexual maturity (7–12 months after hatching) at which point they are evicted from the territory (Grossmann 2007). Tokay geckos are an excellent model to study the effects of the early social life on cognition because offspring can be easily separated and raised alone after hatching or left with their parents to grow up in a family group. In this study, we investigated how individuals reared in social isolation (without interactions with conspecifics) express novel object recognition and space neophobia (the hesitation to approach or total avoidance of a novel stimulus; Crane et al. 2020), exploration, food motivation, habituation (to space and objects) and associative learning as compared to individuals reared in a family group (with interactions with conspecifics, i.e., the parents and siblings). We included measures of behavior because they have been shown to be associated with cognition (Dougherty and Guillette 2018; Kabadayi et al. 2018; van Horik and Madden 2016; Völter et al. 2018). Based on previous work (e.g., Bannier et al. 2017; Janetsian‐Fritz et al. 2018; Munch et al. 2018), we expected individuals raised in social isolation to express lower cognitive ability (decreased ability in novelty recognition, less habituation and lower associative learning ability) due to experiencing fewer social interactions during early life (Humphrey 1976; Jolly 1966).

## 2 Methods

### 2.1 Animals, Breeding Setup and Rearing Conditions

Twenty captive bred tokay geckos ( *Gekko gecko* ), 14 females and 6 males, were included in this study. Sexes were determined by looking at the presence (for males) or absence (for females) of femoral glands (Grossmann 2007). All individuals originated from 10 breeding pairs established from our captive population of 22 adult, captive bred geckos. Across breeding pairs, ten offspring hatched from a first clutch, four from a second clutch, one from a fourth clutch, two from a sixth clutch, two from a seventh clutch, and one each from an eighth, tenth, and eleventh clutch. The distribution of individuals across clutches was based on hatching success (some eggs did not develop and were removed by females). All clutches were incubated within the home enclosure of the parents.

**FIGURE 2:**
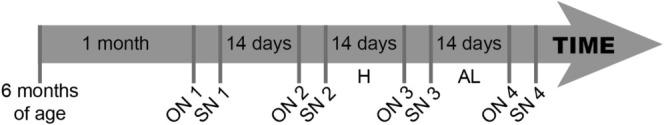
Timeline of the four behavioural experiments. AL, associative learning; H, habituation; ON, object neophobia; SN, space neophobia. Individuals were raised in isolation or in a group for 6 months and then given another month in single housing before the first test.

From around 90 days of incubation, we started checking for hatchlings daily. Offspring started hatching after 78 to 138 days (range) of incubation (dependent on incubation temperature; females hatch at low and males hatch at high temperatures) between May 2022 and March 2023. After hatching, offspring were allocated to either stay within the home enclosure to be raised with their parents or were removed immediately to be raised alone without adults or siblings. Offspring that stayed with their parents either had no siblings or had one or two siblings. Therefore, group sizes ranged from 1 (isolation, *N* = 7 offspring), 3 (adult parents only, *N* = 5 offspring), 4 (adult parents plus one sibling, *N* = 2 offspring) and 5 (adult parents and two siblings, *N* = 6 offspring) individuals. Offspring were raised with parents until they were six months of age and then moved to be housed alone in the same room as hatchlings raised in isolation for one month before the start of testing (Figure 2). This ensured that immediate housing conditions were equally influencing behavior across treatment groups and that potential effects on cognition and behavior were due to long‐term effects of the early social environment (e.g., Bannier et al. 2017; Brandão et al. 2015; Ferreira et al. 2024).

### 2.2 Housing

When raised in isolation, animals were housed in terraria of the size 30 L × 45 B × 45 H cm, made of rigid foam plates with a mesh top, glass front doors as well as hides and enrichment (branches and plants). When raised in groups, animals were housed in terraria of the size 90 L × 45 B × 100 H cm. Except for their size, they are set up exactly the same as the terraria isolated offspring were raised in. An automatic system simulates natural environmental conditions. Animals are exposed to a reversed 12 h:12 h photo period (i.e., light from 6 pm to 6 am, dark from 6 am to 6 pm) to be able to work with them during their natural activity period. For further details on housing and husbandry see the Appendix.

### 2.3 Behavioral Experiments

Testing started one month after individuals were put into single housing (approximately at 7 month of age). All individuals were tested at the same age and tests were done in the same order for all individuals (Figure 2). Therefore, the whole data collection lasted from the 19th of December 2022 until the 7th of November 2023 and all trials were conducted between 8:00 and 15:00.

#### 2.3.1 Object Neophobia (Novel Object Recognition)

Neophobia is the hesitation to approach or total avoidance of a novel stimulus and the result of the cognitive process that allows individuals to distinguish familiar from novel stimuli (Crane et al. 2020; Szabo and Ringler 2023). To reduce stress of handling (Langkilde and Shine 2006) and ensure strong neophobic responses (by avoiding interference of a novel environment; Greenberg and Mettke‐Hofmann 2001), lizards were tested within their home enclosures. At the start of a session, we first placed a dim white light (LED, SPYLUX LEDVANCE 3000K, 0.3 W, 17 lm) on top of the tank mesh lid (lizards expected food when this light was used). Next, a focal individual was located within its enclosure and, if behind a shelter, the shelter was gently removed to expose the lizard for video recording. Thereafter, we presented a cricket in 25 cm long forceps in front of the lizard's snout at a distance of approximately 4–5 cm (optimal attack distance; personal observation) for a maximum of one minute.

**FIGURE 3:**
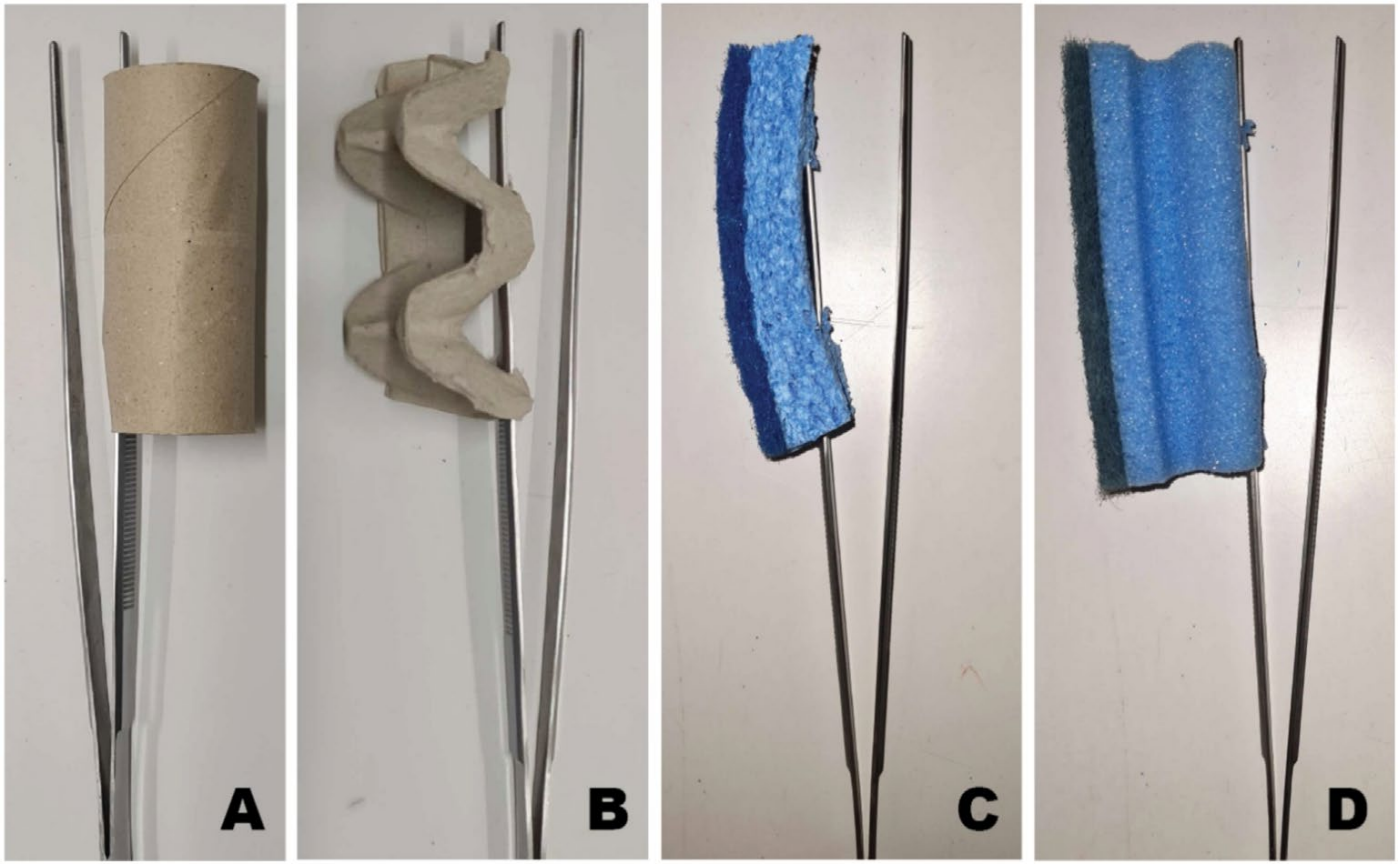
Novel objects used during the object neophobia tests. All objects were attached to 25 cm long forceps and were presented in a random but counterbalanced order across individuals. (A) Toilet paper roll (9.5 cm L, 4 cm diameter; picture taken and modified from Szabo and Ringler 2022), (B) egg carton (9.5 cm L × 4.5 cm H × 4 cm W; picture taken and modified from Szabo and Ringler 2022), (C) course, blue, thin sponge (10 cm L × 2 cm H × 3.8 cm W), and (D) fine, blue, high sponge (11.2 cm L × 4.2 cm H × 3.4 cm W).

Each individual received four sessions of two trials each (test and control) with an inter‐session interval of 14 days (Figure 2) to be able to investigate individual repeatability. In control trials, a single cricket was presented with forceps (same as during regular feeding) while in test trials, the experimenter attached a novel object (toilet paper roll—9.5 cm L and 4 cm diameter; egg carton—9.5 cm L × 4.5 cm H × 4 cm W; fine, blue, high sponge—11.2 cm L × 4.2 cm H × 3.4 cm W; course, blue, thin sponge—10 cm L × 2 cm H × 3.8 cm W; Figure 3) to the forceps next to the cricket. Each object was only used once. The order of presenting test and control trials was randomized but counterbalanced so as to ensure that each individual received the test/control first in two sessions. Furthermore, we randomized the order in which novel objects were presented (in a counterbalanced fashion) as well as the order in which lizards were tested each session to randomize the effects of air temperature on behavior. Trials were recorded using a Samsung S20 smartphone (108 Megapixel, 8K‐FUHD). We measured the time from when the lizard first noticed a cricket (by either moving their head or eyes) until the first strike, regardless of the food being successfully captured or not.

#### 2.3.2 Space Neophobia and Exploration in an Open Field Test

**FIGURE 4:**
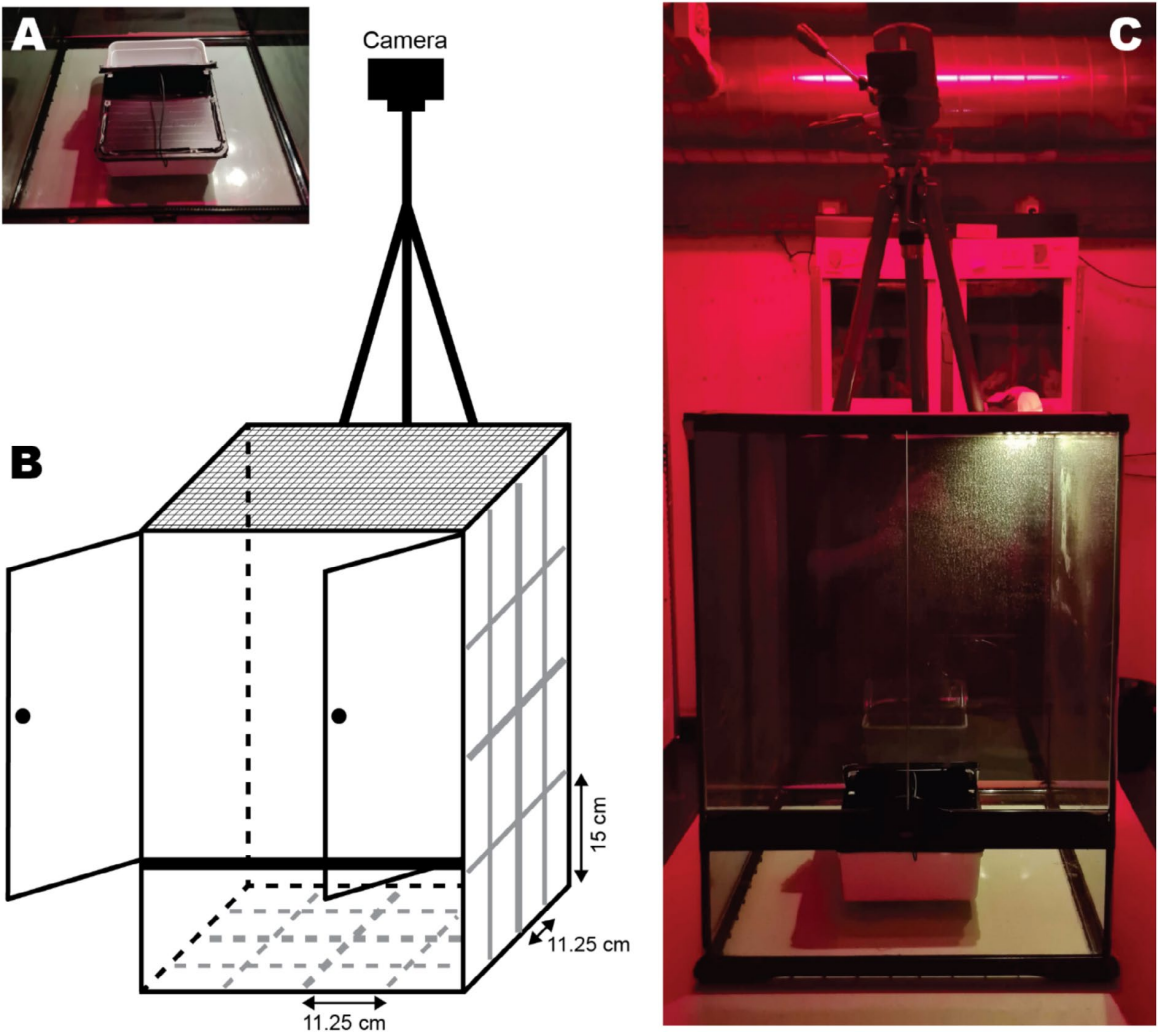
Setup used during the space neophobia test. (A) Picture of the opaque box used to catch lizards (24 cm L × 18 cm W × 7.5 cm H). (B) Schematic representation of the testing tank (45 L × 45 B × 60 H cm) including the camera. The grid painted on all 6 sides of the testing tank to measure exploration is presented in grey. On the long sides, the grid rectangles measured 11.25 × 15 cm. On the bottom and the mesh lid, the grid squares measured 11.25 × 11.25 cm. (C) Picture of the testing tank including the camera mounted on a tripod and the opaque box inside (grid lines not shown). Sides, except for the front and the lid (made out of mesh), were covered in black plastic to make them opaque. Pictures and text taken and modified from Szabo and Ringler (2022, 2023).

Space neophobia tests the hesitation to enter a novel environment, and repeatedly testing individuals reaction to the same environment gives insights into habituation (a short‐term reduction in the response to a stimulus that at least partially reverts back to its original state after a certain period of time with no stimulation; Thorpe 1963; Rankin et al. 2009) to novel space. Lizards were tested in an empty glass terrarium (i.e., testing tank, 45 L × 45 B × 60 H cm, ExoTerra). We used one testing tank, which was placed on top of a table at approximately 100 cm distance, facing (with the front transparent doors) a wall within the animal room. To make the sides and bottom opaque, they were wrapped in black plastic on the outside. To be able to measure exploration, a white grid was drawn onto the outside of the testing tank (grid: 11.25 × 15 cm long sides; 11.25 × 11.25 cm lid and bottom; Figure 4B). To enable video recording in sufficient quality to score animal behavior, we placed a dim white light in the top right corner of the testing tank mesh lid. A GoPro (Hero 8; linear mode, 1080 resolution, 24 FPS) was mounted on a tripod in a way that enabled recording from above (40 cm from the tank lid; Figure 4C).

We first captured a focal lizard by hand and placed it gently in an opaque, plastic box (white opaque bottom of the size 24 cm L × 18 cm W × 7.5 cm H; lid covered in black isolation tape with 6 air holes; Figure 4A). Next, the lizard (within the box) was carefully placed inside the bottom center of the testing tank with the closed box exit facing the back wall (Figure 4C). After 5 min of acclimation, the experimenter started the video recording, opened 1/3 of the box lid carefully, and secured it to the back of the box with a wire to allow the lizard to exit into the testing tank (Figure 4A). Thereafter, the experimenter closed and locked the testing tank door and left the room. Each individual was left undisturbed for 20 min. At the end of the trial, the individual was recaptured by hand and carefully released back into its home enclosure. After each trial, the testing tank and box were thoroughly cleaned with 70% ethanol to remove chemical cues left by each lizard and left for a minimum of 10 min for the alcohol to vanish. Each individual received four trials to investigate individual repeatability (i.e., boldness) and habituation to the testing tank.

#### 2.3.3 Habituation to an Object and Food Motivation

Contrary to the space neophobia test, here we test habituation to a novel object. For five days (Monday to Friday), we presented each individual with an (initially novel) cue card (4 × 4 cm, either depicting a white triangle on a grey background or a black and white stripe pattern, evenly spread across individuals) next to a cricket by attaching the card to 15 cm long forceps using adhesive putty (UHU Patafix) on the back. On a given test day, we first placed a dim white light on top of the enclosure mesh lid. Thereafter, we located the lizard and carefully removed its refuge to expose the lizard for testing if needed. To quantify the change in response, we recorded whether a lizard attacked a cricket presented next to the cue card (1 = yes, 0 = no) across six trials each day (total of 5 × 6 trials = 30 trials). Furthermore, to quantify food motivation, we recorded the number of crickets attacked across all trials. Trials were not recorded on video.

#### 2.3.4 Associative Learning

We define associative learning as an animal associating a stimulus with a reward across repeated exposures (Shettleworth 2009). In this test, we aimed to train lizards to touch a cue card to receive a reward. Similar to the habituation test, lizards received six trials a day for five days. We followed the same procedure as for the habituation test. During the first day, we performed six trials of habituation to ensure that lizards remembered the cue cards after a one‐week break. Thereafter, we presented crickets first in full view of the lizard to draw their attention and next, hid the cricket behind the cue card. We recorded a trial as correct (= 1) if the lizard attacked the cue card. After the attack, we removed the cue card and the lizard received the cricket. If the lizard did not respond after 10 s, we presented the cricket again before hiding it behind the card (this occurred during early stages and in all cases lizards received an “incorrect” score because they did not attack the cue card in these trials). If a lizard attacked the cricket during presentation but not the card, the trial was scored as incorrect (= 0). Associative learning was tested after habituation to ensure that lizards had acclimated to the testing procedure and were familiar with the cards. Trials were not recorded on video.

### 2.4 Video Analysis

We scored videos of object neophobia using the free behavioral coding software BORIS (Friard and Gamba 2016) and measured latencies to an accuracy of 0.001 s. To this end, videos were slowed down to half their speed. If no attack occurred, we recorded occurrence as 0 and assigned this data point a censored latency of 60 s.

From the video of space neophobia, we scored the time taken to exit (exit latency, in seconds) into the novel space (testing tank) starting from when the experimenter locked the testing tank door to when a lizard exited the opaque box by lifting its' tail base over the rim of the box (= exiting with their whole body not counting the tail). If a lizard did not exit the box, we recoded occurrence as 0 and assigned it a censored latency of 1200 s (= 20 min). Furthermore, we also counted the number of times an individual lifted its head out of the box (chin above the rim of the box) before exiting fully as another measure of hesitation to enter the novel environment. To gain a measure that was comparable across individuals and sessions, we divided the number of head lifts by the exit latency (as this latency differed across individuals and sessions). To measure exploration, we counted the number of line crossings after a lizard had exited the box (one line crossing was recorded for exiting the box). If a lizard crossed in a grid corner, we counted two‐line crossings. To accurately estimate each individuals' exploration score we divided the total number of line crossings by the time left for exploration after the opaque box was exited. Because videos could not be scored blind as to test and animal identity, 40% of videos were scored by an observer that was unaware of the objectives of the study and we recorded high inter‐observer reliability (occurrence: Kohens kappa = 1; latency: Spearman rank correlation, *S* = 857.53, *p* < 0.001, *r*_*s*_ = 0.9784056; relative crosses: Spearman rank correlation, *S* = 197.34, *p* < 0.001, *r*_*s*_ = 0.9602133).

### 2.5 Ethical Statement

The experimental procedure applied in this study was strictly non‐invasive and followed the guidelines provided by the Association for the Study of Animal Behavior/Animal Behavior Society for the treatment of animals in behavioral research and Teaching (ASAB Ethical Committee and ABS Animal Care Committee 2023). Experiments were approved by the Suisse Federal Food Safety and Veterinary Office (National No. 33232, Cantonal No. BE144/2020). Captive conditions were approved by the Suisse Federal Food Safety and Veterinary Office (Laboratory animal husbandry license: No. BE4/11). Two offspring died (pathology was inconclusive) during the course of this study, one around 16 weeks and another around 6 weeks after hatching. During pair formation, we monitored adults closely for 12 h to prevent harm. If any aggression occurred within the first hour of pairing, we immediately separated the male and female to avoid injury. Males were then paired with a different female (*N* = 7 attempted pairings total) until we established stable pairs that did not show any aggression towards each other. Similarly, after hatching, we monitored hatchlings that stayed with their parents closely and removed one hatchling (G033) due to concerns of insufficient parental care.

### 2.6 Statistical Analyses

All statistical analyses were run in R version 4.2.2 (R Core Team 2022). We ran Bayesian linear mixed (LMM) and generalized linear mixed models (GLMM) using the package *brms* (Bürkner 2017, 2018, 2021) all including a random effect of animal identity and parent identity (to account for relatedness) except for the models on space neophobia trial 1, in which we removed animal identity as a random effect because the data only included one measure per individual. We used a generic weakly informative normal prior with a mean of 0 and a standard deviation of 1 and ran 4 chains per model of 5000 iterations each and a thinning interval of 1 (default settings). We made sure that model Rhat was 1, that the ESS was above 2000, and checked the density plots and correlation plots to ensure that the models had sampled appropriately. To investigate differences across variable levels (e.g., stimulus) and the results of interactions, we applied estimated marginal means (EMM) *post hoc* tests using the function *emmeans* or *emtrends* from the package *emmeans* (Lenth et al. 2023). We used a test for practical equivalence to determine whether to accept or reject a “null hypothesis”, formulated as “not difference” or “no relationship”, for each fixed effect in a model using the *equivalence_test* function from the package *bayestestR* (Makowski et al. 2019). We report results in which the null hypothesis was accepted (100% within the Region of Practical Equivalence—ROPE) or was undecided as no evidence, and results in which the null hypothesis was rejected (0% within the ROPE) as evidence. Additionally, we provide Bayes factors (BF) to further evaluate the results by determining Bayes Factors from marginal likelihoods using the package *brms*. Bayes factors below 1 indicate no difference, while above 1, BF indicates support for a difference (Schmalz et al. 2023). We report cases in which the equivalence test produced “undecided” results, but Bayes factors were above 1 as evidence. To investigate differences in variance across rearing treatments, we use a two‐tailed *F*‐test using the *var.test* function from base stats. To calculate individual repeatability of behavior, we used the *rptGaussian* function from the package *rptR* (Stoffel et al. 2017). Finally, we used the *corr.test* function from the package *corrplot* (Wei and Simko 2021) to investigate correlations across tests (see Appendix). Due to small sample sizes and imbalanced design (breeding pair identity) we pooled all individuals that were raised socially into a single group called “social” regardless of rearing group size. Data generated during this study and the analysis code are available for download from the Open Science Framework (OSF, https://doi.org/10.17605/OSF.IO/6SP8B).

#### 2.6.1 Object Neophobia (Novel Object Recognition)

First, we subtracted the latency measured in the control trials from the latency measures in the test trial to gain a measure of novelty recognition (negative values indicate longer control latency, while positive values indicate longer test latencies). This difference was then used as the response variable in a Gaussian model with the fixed effects of care (1—raised socially, 0—raised in isolation), stimulus (toilet paper roll, egg carton, low sponge, high sponge), session (1–4), sex (male or female), body condition (SMI—scaled mass index; Peig and Green 2009), temperature (enclosure temperature measured automatically every 15 min) and clutch number (clutch the individual hatched from). We then analyzed differences across stimuli using a *post hoc* EMM test. Furthermore, we compared the variance across rearing treatments based on the average neophobia per individual and calculated agreement repeatability.

#### 2.6.2 Space Neophobia and Habituation to Space

To investigate space neophobia and habituation to novel space, we used two different measures: (1) the censored latency to exit as well as (2) the relative number of times geckos lifted their heads out of the box before exiting. To analyze space neophobia, we used the exit latency in the first trial (response variable) and ran a censored log‐normal model including the fixed effects of care, sex, body condition, temperature, and clutch number. Because we were interested in whether the change in latency across trials (habituation) differed across rearing treatments, we also ran a censored log‐normal model on the latency from all four trials with the addition of a fixed effect of the interaction between care and session. Thereafter, we investigated the result of the interaction using a *post hoc* EMM test. Furthermore, we compared the variance across rearing treatments using the latency for each individual from session 1. Finally, we calculated adjusted repeatability accounting for trials to investigate if geckos showed boldness.

To analyse the relative number of times geckos lifted their head over the rim, we ran Gaussian models (one for session 1 and one on the data from all four sessions) with the same fixed effects as the models for latency. We also used a *post hoc* EMM test to investigate the result of the interaction, compared the variance across rearing treatments using the relative number of head lifts per individual from session 1, and calculated agreement repeatability.

#### 2.6.3 Exploration

To analyze the effects of care, trial, sex, body condition, temperature, and clutch number (fixed effects) on the tendency to move and explore a novel space, we used the relative number of line crossings as the response variable in a Gaussian model. Again, we were interested in whether the change in exploration across sessions differed across rearing treatments by including the interaction between care and trial as a fixed effect. We investigated the result of the interaction using a *post hoc* EMM test and compared the variance across rearing treatments using the average relative number of crosses for each individual. Finally, we calculated adjusted repeatability accounting for session.

#### 2.6.4 Habituation to an Object and Food Motivation

To investigate if lizards habituated to a cue card presented while feeding, we used the occurrence of feeding (1—ate the cricket, 0—did not eat the cricket) as the response variable in a binomial model. We included care, trial (1 to 30), sex, body condition, and temperature as the fixed effects. We were unable to include clutch number as it led to wrong estimates and false results. We were also interested in whether habituation across time differed across rearing treatments by including the interaction between care and trial as an additional fixed effect and investigated the result of the interaction using a *post hoc* EMM test.

To analyze food motivation, we first summed up the trials in which each individual ate a cricket (out of a total of 30 possible trials). We then used this value as the response variable in a Poisson model and included care, sex, body condition, and clutch number as fixed effects. We did not include temperature in this model because we considered all instances of feeding across a whole week of testing. Additionally, as individuals were tested in a different order each day, we assumed that temperature effects would be evenly distributed across days. In addition, we compared the variance across rearing treatments using the number of times a cricket was eaten for each individual.

#### 2.6.5 Associative Learning

To analyze associative learning, we focused on the number of trials in which a lizard showed the desired behavior of first touching the cue card before receiving food. We ran a Poisson model with the number of trials in which the behaviour occurred as the response variable and included the fixed effects of care, sex, body condition, and clutch number. Again, we did not include temperature in this model because we considered all instances of the correct behavior across a whole week of testing. Thereafter, we compared the variance across rearing treatments using the number of times the behavior occurred for each individual.

## 3 Results

### 3.1 Object Neophobia (Novel Object Recognition)

**FIGURE 5:**
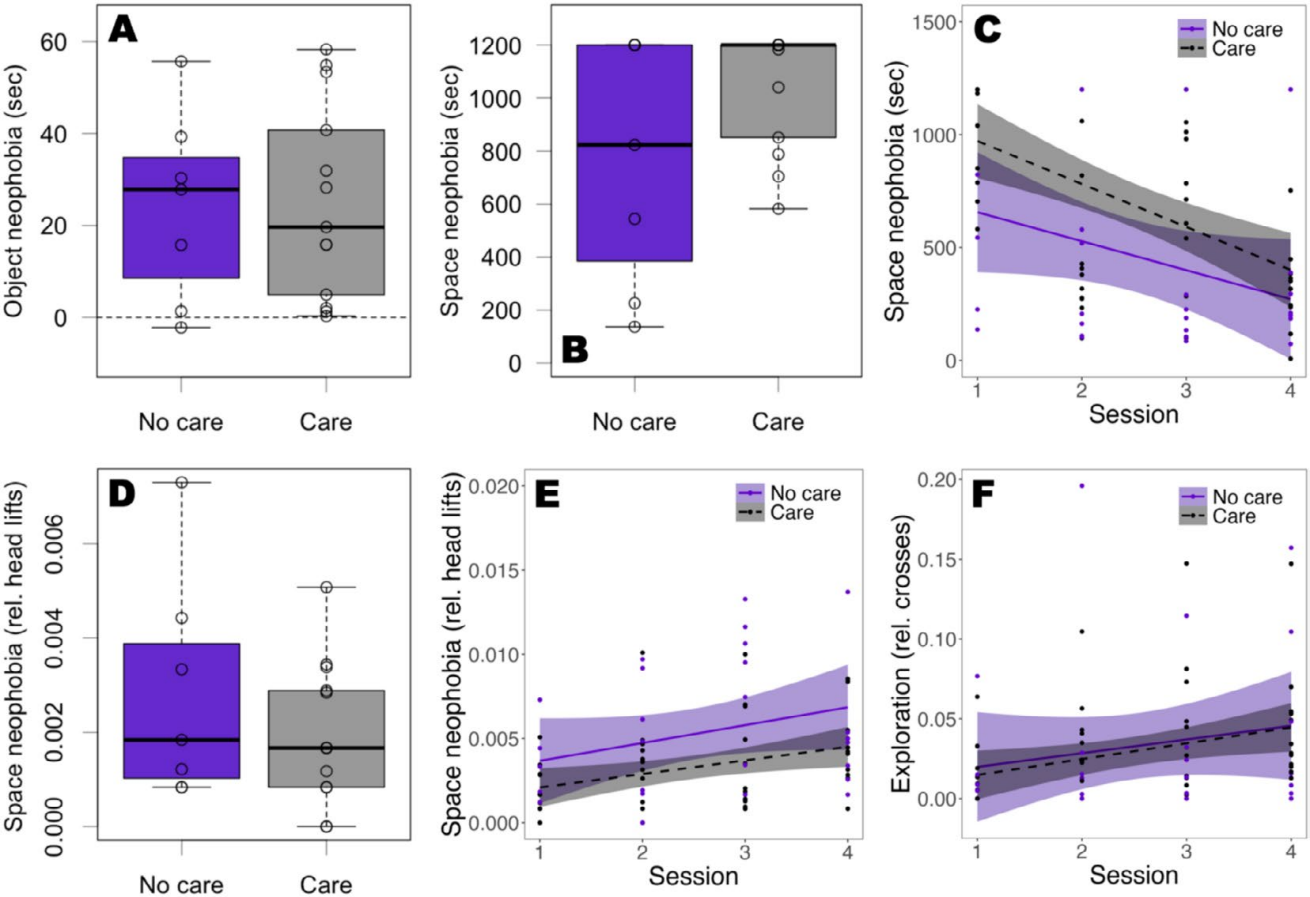
Results from the object and space neophobia test split into rearing treatments (care = family group rearing, *N* = 13, grey; no care = rearing in social isolation, *N* = 7, purple). Points represent individual performance. (A) Average object neophobia (test latency—control latency). The dotted line indicates the same reaction in the control and test trial. The bold line within boxes is the median, the upper box edges are the upper quartile, the lower box edges the lower quartile, the top whisker ends are the maximum and the bottom whisker ends the minimum. (B) Latency to exit into a novel environment (trial 1 only). (C) Predicted latency to exit into the novel environment across sessions (1–4). The shaded area indicates the 95% confidence interval. (D) Relative number of head lifts before exiting into a novel environment (trial 1 only). (E) Predicted relative number of head lifts across sessions. (F) Predicted relative number of crosses across sessions.

Object neophobia was repeatable across all individuals with *R* = 0.405 (CI_low_ = 0.125, CI_up_ = 0.619). We found no evidence that the early social environment (BF = 1.011; Figure 5A), stimulus (BF = 0.985), session (BF = 0.996), sex (BF = 1.014), body condition (BF = 1.006) or temperature (BF = 1.014) had an effect on object neophobia. However, we found a weak relationship of clutch number with object neophobia based on the Bayes factor (BF = 1.917) but this was not confirmed by the test for practical equivalence (null hypothesis was accepted). Furthermore, neophobic responses did not differ across objects used (Table A1). The variance in neophobic responses did not differ across rearing treatments (*F* = 0.946, *p* = 0.997; Figure 5A).

### 3.2 Space Neophobia, Boldness and Habituation to Novel Space

The time taken to exit into a novel environment (space neophobia) was repeatable at *R* = 0.292 (CI_low_ = 0.031, CI_up_ = 0.514) indicating that it is a measure of boldness. We found no evidence that space neophobia (trial 1 responses) differed across rearing treatments (BF = 1.082; Figure 5B), males and females (BF = 0.857), or was associated with body condition (BF = 0.139), temperature (BF = 0.876) or clutch number (BF = 0.436). We found no evidence that the change (across four sessions) in the time taken to exit into a novel environment (habituation to novel space) differed across rearing treatments (EMM, estimate = 0.132, CI_low_ = −0.214, CI_up_ = 0.503, 34.17% inside ROPE). Therefore, we removed the interaction. Based on this simpler model, we found evidence that lizards (regardless of rearing treatment) habituated to the novel environment, shown by a decrease in time to exit across sessions (BF = 3277.987; Figure 5C) and found weak evidence that the time taken to enter a novel space (boldness) differed across rearing treatments (BF = 1.251; Figure 5C). We found no evidence that males differed from females (BF = 0.640), or that body condition (BF = 0.054), temperature (BF = 0.221) and clutch number (BF = 0.081) were related to the time taken to exit when all four sessions were taken into account (Table A2). The variance in space neophobia differed across rearing treatments, with individuals from the no care group showing higher variation than individuals that received care (*F* = 4.199, *p* = 0.033).

Similarly, the relative number of head lifts before exit was repeatable at *R* = 0.298 (CI_low_ = 0.042, CI_up_ = 0.510) indicating that it is also a measure of boldness. We found no evidence that the relative number of head lifts in the first trial differed across rearing treatments (BF = 0.007; Figure 5D), sexes (BF = 0.002), or was related to body condition (BF = 0.0001), temperature (BF = 0.002) or clutch number (BF = 0.003; Table A2). Furthermore, we found no evidence that the change in the relative number of head lifts differed across rearing treatments (EMM, estimate = 0.0002, CI_low_ = −0.0009, CI_up_ = 0.0013, 100% inside ROPE) when all four sessions were taken into account. Therefore, we removed the interaction. This simpler model produced no evidence of an effect of rearing treatment (BF = 0.013; Figure 5E), session (BF = 0.070, although the test for practical equivalence indicated a relationship; Figure 5E), sex (BF = 0.001), body condition (BF = 0.0001), temperature (BF = 0.0004) or clutch number (BF = 0.002) on the relative number of head lifts before exiting (Table A2). The variance in the relative number of head lifts before exit did not differ across rearing treatments (*F* = 2.578, *p* = 0.154).

### 3.3 Exploration

The relative number of crosses was repeatable at *R* = 0.680 (CI_low_ = 0.429, CI_up_ = 0.818). We found no evidence that the change in the relative number of line crossings differed across rearing treatments (EMM, estimate = −0.001, CI_low_ = −0.011, CI_up_ = 0.009, 100% inside ROPE). Therefore, we removed the interaction. This simpler model showed evidence that exploratory behavior increased across sessions (BF = 5.419; Figure 5F), while we found no evidence that rearing treatment (BF = 0.035; Figure 5F), sex (BF = 0.026), body condition (BF = 0.001), temperature (BF = 0.006), nor clutch number (BF = 0.006) were associated with exploratory behavior (Table A3). We found no evidence that the variance in the relative number of crosses differed across rearing treatments (*F* = 2.963, *p* = 0.103).

### 3.4 Habituation and Food Motivation

**FIGURE 6:**
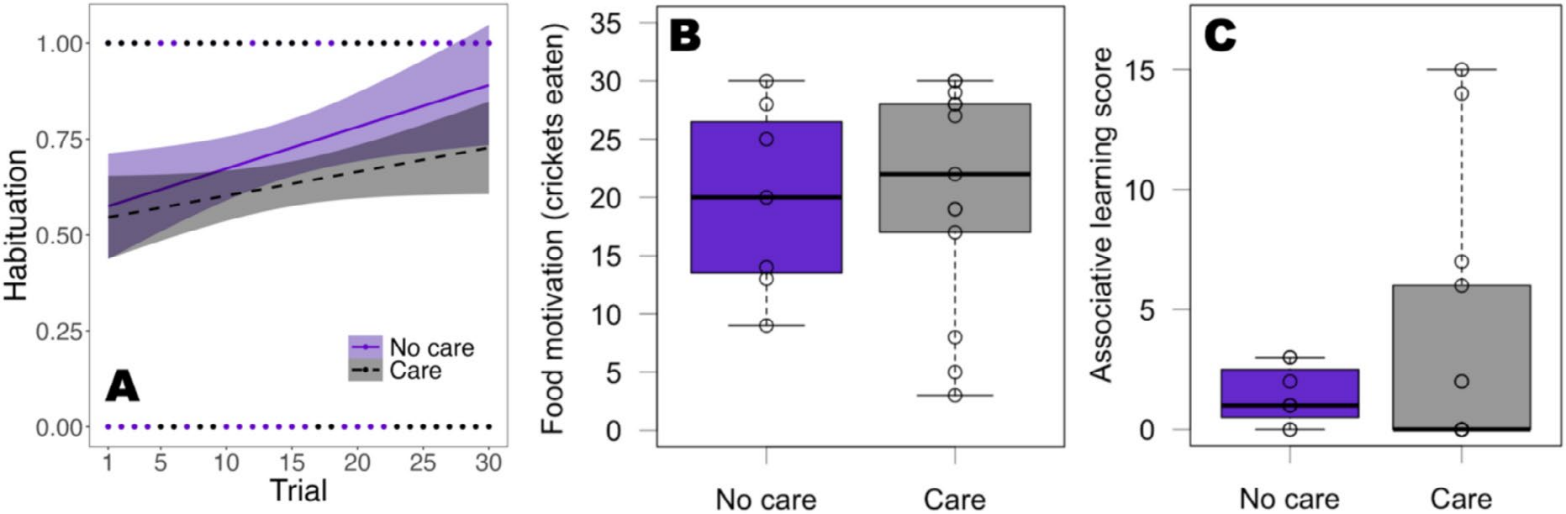
Results from the habituation and associative learning test split into rearing treatments (care = family group rearing, *N* = 13, grey; no care = rearing in social isolation, *N* = 7, purple). Points represent individual responses. (A) Predicted probability to attack a cricket next to a novel cue card across trials. The shaded area indicates the 95% confidence interval. (B) Number of crickets consumed. (C) Number of trials in which an individual touched the cue card to receive a reward. The bold line within boxes is the median, the upper box edges are the upper quartile, the lower box edges the lower quartile, the top whisker ends are the maximum and the bottom whisker ends the minimum.

We found no evidence that the change in the likelihood to eat next to a cue card (i.e., habituation) differed across rearing treatments (EMM, estimate = 0.037, CI_low_ = −0.036, CI_up_ = 0.111, 97.13% inside ROPE). Therefore, we removed the interaction. This simpler model revealed no evidence that habituation occurred across trials (BF = 0.037; Figure 6A). Furthermore, we found no evidence that rearing treatment (BF = 0.754; Figure 6A), sex (BF = 1.105), body condition (BF = 0.109) nor temperature (BF = 1.069) were associated with habituation (Table A4).

Similarly, we found no evidence that rearing treatment (BF = 0.438; Figure 6B), sex (BF = 0.586), body condition (BF = 0.025) or clutch number (BF = 0.059) influenced how many crickets lizards ate during the habituation test (Table A5). We found no evidence that the variance in the relative crickets eaten differed across rearing treatments (*F* = 0.699, *p* = 0.689).

### 3.5 Associative Learning

We found evidence that the variance in the number of correct trials did differ across rearing treatments (*F* = 0.055, *p* = 0.002; Figure 6C), with a higher variance in animals raised with parents. However, there was no evidence that the rearing treatment (BF = 0.904; Figure 6C), sex (BF = 0.875), body condition (BF = 0.071), or clutch number (BF = 0.175) influenced the number of correct trials (Table A6).

## 4 Discussion

The aim of our study was to test how being able to interact with conspecifics during early life or the lack of interactions (social isolation rearing) influences the development of cognition and behavior in a social lizard (e.g., Bannier et al. 2017; Schäble et al. 2007; Zhang 2017). Confirmation of improved cognitive ability due to multifaceted early social experiences would provide evidence that complex social environments can facilitate cognitive evolution (social intelligence hypothesis; Humphrey 1976; Jolly 1966; Chance and Mead 1953). We investigated novelty recognition through object neophobia, habituation to novel objects and space as well as associative learning and expected to find lower cognitive abilities in animals that were raised in social isolation compared to animals raised within a family group due to the lack of social stimulation early in life. Overall, we found that individuals that were raised in a social group showed lower variation in space neophobia and higher boldness measured as the time taken to enter a novel space and expressed a larger variation in associative learning ability. However, the average associative learning ability across social rearing treatments did not differ statistically. We also found habituation to novel space shown by a decrease in the latency to enter the novel space and an increase in exploration across trials regardless of early social experiences. In no case was food motivation, body condition, temperature or clutch number associated with any cognitive measure taken in our experiment.

We found that the early social environment influenced only some but not all of our behavioral and cognitive measures. Lizards raised in social isolation were bolder (entered an novel environment faster across four trials) and showed larger variation in space neophobia compared to lizards raised in a family group. Higher space neophobia (or being shyer) could provide advantages when it comes to delaying dispersal. An unwillingness to enter novel space, as shown by the social treatment group, might be a direct result of parental care to prolong the benefits of protection until forced to disperse by the parents. This could also explain the lower variation in responses in the care group due to the general benefit of staying under parental protection for as long as possible. Alternatively, being raised in a deprived environment might have increased isolated individuals' novelty seeking behavior. However, this seems unlikely because we found no differences in object neophobia based on the early social environment.

We also found larger variation in associative learning by individuals from the social rearing treatment but no average difference between the groups, meaning that some individuals from the social rearing treatment far outperformed others within both the social and isolation rearing treatments. Therefore, social rearing induced changes in cognition in some individuals but not all. Enhanced learning ability might give some individuals a competitive advantage over others including siblings. For example, enhanced learning ability is related to increased reproductive success (e.g., Ashton et al. 2018; Smith et al. 2015; White et al. 2022) and survival (e.g., Dayananda and Webb 2017; Madden et al. 2018) although this relationship might depend on other factors (e.g., mating tactic, incubation temperature, or body condition) and not all studies find such a relationship between cognition and fitness measures (e.g., Huebner et al. 2018). Furthermore, better learning ability in the context of foraging might help individuals to occupy different social niches and avoid competition for resources especially with related individuals later in life (Humphrey 1976; Montiglio et al. 2013). However, as our experiment was performed in captivity we do not know if these few “smarter” individuals would fare better as predicted by the social intelligence hypothesis. Studies linking cognition and survival are still scarce (Rochais et al. 2022) but important to understand how the link between sociality and cognition might play out on an evolutionary scale; if “smarter” individuals indeed survive longer and produce more offspring. More generally, our sample size was low, and therefore, our power to detect differences was diminished. Additionally, further variation in behavior might have been introduced by differences in parental age (e.g., Ronce et al. 1998), incubation time (e.g., Sakata and Crews 2003) and hatching order (e.g., Campbell et al. 2017). Therefore, we might have only been able to detect the strongest effects while other, more subtle influences were masked by individual variation. Furthermore, even though geckos were raised in differently sized family groups, we were unable to analyze performance separately for these different groups due to the low number of replicates (e.g., one family with two offspring and two families with three offspring). To gain a better understanding of the subtle influence of early social experiences on the development of cognition, future studies should include larger sample sizes across a broader range of social environmental treatments.

Previous studies in lizards have shown mixed results as to the influence of the early social environment on cognition, potentially due to the large variation in social environments tested (e.g., siblings versus parents). Tree skinks (*Egernia striolata*) raised with an age‐matched, unrelated partner did not differ in their spatial learning ability in a vertical maze compared to individuals raised alone (Riley et al. 2017). Furthermore, both socially reared and isolated individuals learned a discrimination and reversal task, with individuals from both groups similarly likely to use social information from a demonstrator (Riley et al. 2018). Conversely, White's skinks (*Liopholis whitii*) reared with their mother showed better learning to escape a simulated predator attack by decreasing errors across trials compared to skinks raised in social isolation that did not decrease errors (Munch et al. 2018). In the present study, we find differences in space neophobia and associative learning in some individuals across rearing treatments. One striking variation across these and our study is that when offspring were raised with adults (mother or both parents) we find an influence on cognitive development, while when they are raised with age matched conspecifics there is no effect. Similarly, a study in the cooperatively breeding cichlid fish, *Neolamprologus pulcher* , found that the presence of older group members during the early life decreased object neophobia (Bannier et al. 2017). It is possible that, depending on the social expression of a species, the presence of certain conspecifics such as the parents exerts a stronger influence than other individuals (e.g., siblings). Future studies in the tokay gecko should, therefore, compare the effects of parents compared to age matched social partners. Furthermore, tokay geckos are part of the Gekkonidae family, as opposed to tree skinks and White's skinks, which are part of the Scincidae family (Pyron et al. 2013), and consequently, the results of our study provide important new insights from a broader phylogenetic perspective within lizards.

Our study and many others investigating how sociality is linked to the development of cognition often test general cognitive abilities such as associative learning, discrimination and reversal learning, spatial learning or neophobia (e.g., Brandão et al. 2015; Meagher et al. 2015; Riley et al. 2017). Even though some studies have found an effect of sociality on non‐social cognitive abilities (e.g., Ashton et al. 2018), arguably, we would expect the most pronounced effect to occur in the social domain such as during social learning or when using social information to make decisions. Indeed, a study across six lemur species demonstrated that group size predicted cognitive performance only in social (perspective taking) but not non‐social cognitive tests (inhibitory control; MacLean et al. 2013). Furthermore, a recent study in the cichlid fish, *N. pulcher* , showed an effect of the early social environment on behavioral flexibility only in social contexts but not in non‐social contexts (Ferreira et al. 2024). In contrast, tree skinks were similarly unlikely to use social information during social learning, regardless of the social environment during rearing (Riley et al. 2018). In the current study, we were unable to include social cognitive tests due to time constraints. To gain a truly comprehensive understanding of how sociality influences cognition, future studies should test a wide range of cognitive abilities, both social and non‐social.

Object and space neophobia as well as boldness and exploration are commonly investigated animal personality traits (animal personality is defined as consistent individual differences across time and/or contexts; Carere and Locurto 2011) and personality has been linked to cognition (Carere and Locurto 2011) and can be influenced by early social life experiences (e.g., Edenbrow and Croft 2013; Haller et al. 2014; Liedtke et al. 2015). Therefore, we included some non‐cognitive measures to determine if early social life experiences influence cognition directly, or indirectly through non‐cognitive factors. Generally, we found that only a few behavioral and cognitive measures correlated (see Appendix), but, in most cases, measures collected in the same test were correlated, suggesting that they were not independent. Nonetheless, this indicates that the early social environment affected cognition directly. However, even though we raised offspring in the same environment for one month before testing (Ferreira et al. 2024; Taborsky 2017), long‐lasting effects of differences in the physical environment (e.g., enclosure size) that were necessary to accommodate a larger number of individuals could have also affected behavior and cognitive development. This should be accounted for in future studies.

Object neophobia, both measures of space neophobia and exploration were repeatable in our study indicating the presence of personality traits, of which one, boldness, was influenced by early‐life experiences. Such developmental effects on personality were previously shown in mammals (e.g., Haller et al. 2014), fish (e.g., Edenbrow and Croft 2013), amphibians (e.g., Płaskonka et al. 2024) and spiders (e.g., Liedtke et al. 2015). Interestingly, overall, repeatability of behavior was higher in offspring than in their parents (measures from parents: *R*_object_ = 0.124; *R*_space_ = 0.044; *R*_exploration_ = 0.538; Szabo and Ringler 2022, 2023). On average, studies on novel object tests find repeatability of 0.47 (Takola et al. 2021) while studies on behavior find on average a repeatability of 0.37 (Bell et al. 2009). Therefore, the values we find in the current study are within the range of what would be expected. What is more interesting is the increased repeatability in the individuals tested in the current study, which might have a number of causes. First, for space neophobia and exploration, we might have been able to estimate repeatability more reliably in the current study because we used four instead of two repetitions. However, this explanation cannot account for the increased repeatability in object neophobia because we used four repetitions previously. Second, animals in our study were between 7 and 9 months old, whereas adults were between 2 and 6 years old. Age might, therefore, be a factor influencing repeatability. Contrary to our results, a study in turtles ( *Terrapene carolina* ) showed no difference in the magnitude of repeatability in boldness between adults and juveniles, which was stable across years (Carlson and Tetzlaff 2020). Similarly, a study in zebra finches ( *Taeniopygia guttata* ) showed that activity, aggression, and exploration were repeatable across life stages; boldness was not (Wuerz and Krüger 2015). Overall, we still have an incomplete understanding about how personality develops and is maintained across an individual's lifetime, a gap that future research needs to fill (Cabrera et al. 2021).

## 5 Conclusions

The early social environment experienced after hatching had no effect on most measures of non‐social cognition tested in our facultatively social gecko. However, we did find larger variation in associative learning, showing better performance of some socially reared individuals and higher space neophobia in the care group. Geckos, and more generally lizards, provide exciting albeit underutilized models to investigate the relationship between sociality, behavior, and cognition, especially considering that they are facultative social with independent offspring. Consequently, by testing different species expressing a range of social complexity, we might gain a unique perspective into which cognitive abilities could have been selected for during the early stages in the evolution of sociality and provided an adaptive advantage to mitigate the challenges of group living.

## Author Contributions

**Birgit Szabo:** conceptualization (lead), data curation (lead), formal analysis (lead), investigation (lead), methodology (lead), project administration (lead), resources (equal), validation (lead), visualization (lead), writing – original draft (lead), writing – review and editing (equal). **Eva Ringler:** funding acquisition (lead), resources (equal), writing – original draft (supporting), writing – review and editing (equal).

## Conflicts of Interest

The authors declare no conflicts of interest.

## Data Availability

The datasets generated and analyzed during the current study as well as the code used for analysis are available in the Open Science Framework repository https://doi.org/10.17605/OSF.IO/6SP8B.

## Additional Methodological Details

### Breeding

All adults were purchased from different breeders and were between 3 and 8 years old (only one female was 8 years old, all others were between 3 and 4 years old). Adults were paired in January 2022 and stayed in pairs until January 2023. Females produced their first clutches in February 2022 and continued to produce clutches approximately every 30 days. All eggs and their location were recorded upon discovery of a clutch.

### Housing

Terraria are fitted with a compressed cork wall fixed to the back, cork branches cut in half hooked on the back (functioning as shelters), cork branches allowing lizards to climb, and life plants as enrichment. Each terrarium has a drainage layer of expanded clay, separated by a mosquito mesh from the soil placed on top (organic tropical forest soil; Dragon BIO‐Ground). We spread sphagnum moss and autoclaved red oak leaves on the soil as shelter and food for the isopods that decompose the faecal material of the lizards. Terraria are organised on shelves in three layers. The environmental system imitates sunrise and sunset, which are accompanied by changes in temperature reaching approximately 25°C during night and 31°C during day. In addition, an UVB light (Exo Terra Reptile UVB 100, 13 W) is provided on top of the terraria during the day. A red light (PHILIPS TL‐D 36W/15 RED) invisible to geckos (Loew 1994) is kept on for 24 h so as to enable experimenters to work with the lizards. Furthermore, lizards can thermoregulate to their optimal body temperature at any time due to a heat mat (TropicShop) attached to the right outer wall of each enclosure, which locally increases the temperature by 4°C–5°C. Humidity is kept at 50%, but every 12 h, at 5 pm and 4 am, 30 s of rainfall (with reverse osmotic water) briefly increases humidity to 100%.

### Husbandry

Offspring were fed five times per week, with 10–15, small to medium sized house crickets ( *Acheta domesticus* ) using scatter feeding. The size of the crickets was adjusted to the changing head size while growing. Offspring at about 3–4 month of age and adult geckos are fed 3–5, adult house crickets using 25 cm long forceps in order to control food intake. To provide optimal nutrition to our animals (vitamin D and calcium), the insects are fed with high protein dry cat food (various brands), cricket mix (reptile planet LDT), and fresh carrots. Fresh water for geckos is supplied ad libitum in water bowls (to prevent small offspring from drowning, we added a large stone to ensure easy). Moreover, adult geckos are weighed (±1 g) every month and measured (SVL—snout vent length, ±0.5 cm) approximately every 3 months, to track their body condition. Offspring were measured (SVL—snout vent length, ±0.5 cm) every 2 weeks until they reached 6 months of age after which they were put on the same monitoring schedule as adults.

### Test Sequence

Object neophobia was tested on Mondays to coincide with a feeding day after lizards had not received food for 2 days over the weekend (Figure 1). Space neophobia was tested the following Tuesday (which was a non‐feeding day; Figure 1). Habituation was tested between the second and third object/space neophobia session, while associative learning was tested between the third and fourth object/space neophobia session (Figure 1; except for four individuals [G039, G040, G041, G042] which were tested for habituation and associative learning after object/space neophobia testing had finished due to logistic reasons). The order of tests was chosen due to logistical reasons and minimise the testing period. If possible, tests were conducted within the home enclosure of animals to reduce stress of handling and exposure to new environments (except for the space neophobia test which was a test to measure responses to novel space; Langkilde and Shine 2006).

## Supplementary Analyses and Results

### Association Between Test Performances

To understand if performance across tests was related within individuals, we performed pairwise Spearman rank correlations with a Holm correction for multiple testing. From the object neophobia test, we included the average difference in attack latency for each individual. From the space neophobia test, we included the latency and relative number of head lifts in the first session, the difference in latency to exit from the first to the last session (as a measure of habituation), and the average number of relative crosses for each individual (for exploration). Finally, from the habituation test, we included the difference in the number of attacks from session one to five (as another measure of habituation), and the number of crickets eaten, and from the associative learning test, we included the number of correct trials for each individual.

Spearman rank correlations showed that the latency to exit was negatively correlated with the number of head lifts (*r*_*s*_ = −0.72), which indicates that more neophobic individuals that take longer to exit into the novel environment lift their heads less often before exiting. Furthermore, exploration was negatively correlated with the latency to exit (*r*_*s*_ = −0.59) and positively correlated with the number of head lifts (*r*_*s*_ = 0.79). This indicates that more neophobic individuals were less exploratory. We also found that object neophobia was positively correlated with the number of head lifts (*r*_*s*_ = 0.73) demonstrating that individuals that were more neophobic towards novel space were less neophobic towards novel objects. Object neophobia was positively correlated with exploration (*r*_*s*_ = 0.69); individuals with lower object neophobia explored less. Finally, the latency to exit was negatively correlated with habituation to a novel cue card (*r*_*s*_ = −0.51), indicating that individuals that took longer to enter a novel environment habituated more to a novel object. No other measures were correlated above a coefficient of 0.5 (Table A7).

**TABLE A1:** Estimates and test statistics from the model and post hoc test analysing the behaviour shown during the object neophobia test.

Parameter	Estimate	95% CI_low_	95% CI_up_	% inside ROPE
Intercept	42.936	−36.529	121.800	3.12
Care yes	−0.046	−1.975	1.905	100
High sponge	0.015	−1.947	1.948	100
Low sponge	0.033	−1.887	1.652	100
Toilet paper roll	−0.068	−2.036	1.888	100
Session	0.082	−1.848	2.001	100
Male	−0.134	−2.057	1.813	100
Body condition	−0.441	−1.304	0.434	100
Temperature	−0.250	−2.267	1.730	100
Clutch number	−1.065	−2.576	0.458	100

Difference	Post hoc test results			
	Estimate	95% CI_low_	95% CI_up_	% inside ROPE
Egg carton—high sponge	−0.009	−1.95	1.93	7.93
Egg carton—low sponge	−0.028	−1.98	1.84	8.42
Egg carton—toilet paper roll	0.060	−1.88	2.04	8.85
High sponge—low sponge	−0.023	−2.75	2.69	5.67
High sponge—toilet paper roll	0.090	−2.74	2.80	5.47
Low sponge—toilet paper roll	0.107	−2.67	2.82	5.76

Abbreviations: CI, credible interval; ROPE, region of practical equivalence.

**TABLE A2:** Estimates and test statistics from the models analysing the behaviour shown during the space neophobia test.

Parameter	Estimate	95% CI_low_	95% CI_up_	% inside ROPE
*Latency to exit*				
Trial 1				
Intercept	−11.441	−57.401	32.918	0.05
Care yes	0.284	−1.628	2.002	0.81
Male	−0.112	−1.785	1.670	1.02
Body condition	0.095	−0.173	0.543	6.40
Temperature	0.352	−1.293	1.798	0.79
Clutch number	−0.227	−0.934	0.599	1.75
All 4 trials				
Intercept	7.791	−2.789	17.983	0.07
Care yes	**0.694**	**−0.502**	**1.839**	**0.61**
Session	**−0.478**	**−0.681**	**−0.285**	**0.00**
Male	0.379	−0.764	1.506	1.12
Body condition	0.026	−0.068	0.105	15.29
Temperature	−0.137	−0.492	0.225	3.51
Clutch number	0.010	−0.195	0.200	8.92
*Relative number of heat lifts*				
Trial 1				
Intercept	0.0312	−0.0137	0.0732	0.26
Care yes	−0.0026	−0.0053	0.0003	2.12
Male	0.0006	−0.0023	0.0036	10.12
Body condition	0.0001	−0.0002	0.0002	97.89
Temperature	−0.0011	−0.0027	0.0005	7.02
Clutch number	0.0001	−0.0005	0.0005	53.34
All 4 trials				
Intercept	0.0150	−0.0123	0.0423	1.09
Care yes	−0.0037	−0.0072	−0.0001	0.97
Session	0.0009	0.0004	0.0014	0.00
Male	−0.0006	−0.0039	0.0028	15.99
Body condition	0.0001	−0.0002	0.0002	100
Temperature	−0.0004	−0.0014	0.0006	38.35
Clutch number	−0.0001	−0.0007	0.0005	78.85

*Note:* Significant results are highlighted in bold.

Abbreviations: CI, credible interval; ROPE, region of practical equivalence.

**TABLE A3:** Estimates and test statistics from the model analysing exploratory behaviour during the space neophobia test.

Parameter	Estimate	95% CI_low_	95% CI_up_	% inside ROPE
Intercept	0.0034	−0.307	0.320	2.16
Care yes	−0.0173	−0.076	0.042	9.95
Session	**0.0096**	**0.005**	**0.014**	**0.00**
Male	0.0034	−0.055	0.060	12.61
Body condition	0.0004	−0.003	0.004	100
Temperature	0.0001	−0.009	0.009	64.91
Clutch number	−0.0035	−0.012	0.006	53.71

*Note:* Significant results are highlighted in bold.

Abbreviations: CI, credible interval; ROPE, region of practical equivalence.

**TABLE A4:** Estimates and test statistics from the model analysing habituation.

Parameter	Estimate	95% CI_low_	95% CI_up_	% inside ROPE
Intercept	−21.258	−50.337	7.493	0.35
Care yes	−0.075	−1.535	1.371	21.25
Trial	0.020	−0.011	0.053	100
Male	0.650	−0.824	2.163	14.03
Body condition	0.069	−0.054	0.208	97.13
Temperature	0.625	−0.418	1.690	14.66

Abbreviations: CI, credible interval; ROPE, region of practical equivalence.

**TABLE A5:** Estimates and test statistics from the model analysing food motivation during the habituation test.

Parameter	Estimate	95% CI_low_	95% CI_up_	% inside ROPE
Intercept	2.014	−1.601	5.485	2.27
Care yes	0.062	−0.719	0.815	22.23
Male	0.302	−0.508	1.093	14.88
Body condition	0.010	−0.041	0.062	100
Clutch number	−0.026	−0.152	0.098	91.01

Abbreviations: CI, credible interval; ROPE, region of practical equivalence.

**TABLE A6:** Estimates and test statistics from the model analysing the number of correct trials performed during the associative learning test.

Parameter	Estimate	95% CI_low_	95% CI_up_	% inside ROPE
Intercept	−0.162	−9.470	9.166	1.82
Care yes	0.366	−1.200	1.935	9.94
Male	0.105	−1.486	1.631	10.65
Body condition	−0.008	−0.146	0.128	90.01
Clutch number	0.079	−0.234	0.406	45.02

Abbreviations: CI, credible interval; ROPE, region of practical equivalence.

**TABLE A7:** Correlation matrix including both correlation coefficients and *p*‐values across all measures taken during the whole experiment.

	Object neophobia	Latency to exit	Head lifts	Habituation to novel space	Exploration	Habituation to a stimulus card	Food motivation	Correct trials
Object neophobia	*r*_*s*_ = 1	*r*_*s*_ = −0.33	***r***_***s***_ **= 0.73**	*r*_*s*_ = −0.04	***r***_***s***_ **= 0.69**	*r*_*s*_ = −0.41	*r*_*s*_ = −0.13	*r*_*s*_ = −0.23
Latency to exit		*r*_*s*_ = 1	***r***_***s***_ **= −0.72**	*r*_*s*_ = −0.11	***r***_***s***_ **= −0.59**	***r***_***s***_ **= −0.51**	*r*_*s*_ = 0.08	*r*_*s*_ = 0.24
Head lifts			*r*_*s*_ = 1	*r*_*s*_ = 0.10	***r***_***s***_ **= 0.79**	*r*_*s*_ = −0.19	*r*_*s*_ = −0.25	*r*_*s*_ = −0.27
Habituation to novel space				*r*_*s*_ = 1	*r*_*s*_ = 0.16	*r*_*s*_ = 0.10	*r*_*s*_ = −0.04	*r*_*s*_ = −0.19
Exploration					*r*_*s*_ = 1	*r*_*s*_ = −0.16	*r*_*s*_ = −0.04	*r*_*s*_ = −0.39
Habituation to a stimulus card						*r*_*s*_ = 1	*r*_*s*_ = 0.16	*r*_*s*_ = 0.06
Food motivation							*r*_*s*_ = 1	*r*_*s*_ = 0.46
Correct trials								*r*_*s*_ = 1

*Note:* *r*_*s*_, Spearman rank correlation coefficient. All *p*‐values are corrected for multiple testing (Holm correction). Significant correlations are highlighted in bold. Shaded areas are the correlations of each variable with itself.
